# Exploration of the Specificity of Motor Skills Hypothesis in 7–8 Year Old Primary School Children: Exploring the Relationship Between 12 Different Motor Skills From Two Different Motor Competence Test Batteries

**DOI:** 10.3389/fpsyg.2021.631175

**Published:** 2021-06-18

**Authors:** Hermundur Sigmundsson, Karl M. Newell, Remco Polman, Monika Haga

**Affiliations:** ^1^Department of Psychology, Norwegian University of Science and Technology, Trondheim, Norway; ^2^Education and Mindset Research Centre, University of Iceland, Reykjavik, Iceland; ^3^Department of Kinesiology, University of Georgia, Athens, GA, United States; ^4^School of Exercise and Nutrition Sciences, Queensland University of Technology, Brisbane, QLD, Australia; ^5^Department of Teacher Education, Norwegian University of Science and Technology, Trondheim, Norway

**Keywords:** assessment, coordination, motor competence, specificity, children

## Abstract

This study examined the specificity hypothesis by examining the association between two specific motor competence test batteries [Movement Assessment Battery for Children (MABC) and Test of Motor Competence (TMC)] in a sample of young children. In addition, we explored the factorial structure of the MABC and TMC. A total of 80 children participated in the study (38 girls and 42 boys) with a mean chronological age of 7.9 years (SD 0.55). The correlation between total score MABC and total z-score TMC was *r* = 0.46. In general, low pair-wise correlations (*r*^2^ < 0.20) between the different motor tasks were found. The highest correlation was between the placing bricks and building bricks *r* = 0.45 (TMC); the stork balance and jumping in squares *r* = 0.45 (MABC). These low pair-wise relations of items are consistent with findings from younger and older children's age-related motor competence test batteries. Principal components analysis (PCA) showed that the 1st component accommodated 25% of the variance and was dominated in the top five variable weightings by items of the MABC test; whereas the 2nd component accommodated 12% of the variance with the higher weightings all from the TMC test. The findings provide evidence with children for specificity rather than generality in learning motor skills a viewpoint that has predominantly been driven by adult learning studies. The PCA revealed that the MABC and TMC are testing different properties of children's motor competence though in both cases the variance accounted for is relatively modest, but generally higher than the motor item pair-wise correlation.

## Introduction

Children participate in a host of physical activities that require co-ordination of the motor system, including activities of daily living, play, sport and academic tasks. To do so, children need basic motor competence to handle these practical everyday motor skills (Vedul-Kjelsås et al., 2012). Motor competence (MC) can be conceived as a person's level of performance on one or more specific skills or different motor acts, including coordination of both fine and gross motor skills that are necessary to participate and function effectively in everyday life (Henderson and Sugden, 1992). Having a well-developed movement repertoire seems particularly important for children to engage in regular physical activity, exercise and sport (Holfelder and Schott, 2014). Moreover, high levels of MC in children has been associated with increased self-esteem, physical fitness, higher peer group status and popularity, and enhanced cognitive functioning (Jansen et al., 2011; Cattuzzo et al., 2016; Schmidt et al., 2017).

There is currently no agreement about what the MC construct encompasses (Rudd et al., 2015, 2016) and there is no “gold-standard” on how to measure it (Henderson and Barnett, 1998; Crawford et al., 2001; Bardid et al., 2019). Difficulties with constructs and the motor tasks that are used to operationalize those constructs limit the development of improved assessment tools for MC (Larkin and Cermak, 2002; Hulteen et al., 2020). Indeed, there is a plethora of tests or test batteries for assessing MC. These test batteries include a variety of test items, apply different procedures for scoring performance such as product or process-orientation, and are designed for different purposes. Several test batteries have been developed to identify children with mild to moderate motor coordination difficulties or general developmental delays [The Movement Assessment Battery for Children (MABC) and Test of Gross Motor Development–second edition (TGMD-2)], while other test batteries monitor children's general movement capabilities over time (Canadian Agility and Movement Skill Assessment, Longmuir et al., 2015). Additionally, researchers from different countries seem to prefer different tests for assessing movement competence in children (Bardid et al., 2015; Rudd et al., 2016). This has generated considerable uncertainty in the interpretation and application of the various test results across practical and research-contexts.

Common characteristics of test batteries for MC typically measure various aspects of speed, accuracy, sureness, coordination of the two hand, hand-eye coordination, hand foot coordination and/or static/dynamic balance (e.g., Henderson et al., 2007) often categorized within the higher order dimensions of locomotor, object control and stability (Gallahue et al., 2012). In addition to this, the quality or outcome of the execution of the skill might depend on the context in which it is performed (Newell, 1986; Sigmundsson et al., 2017).

In addition, measurements of MC should be easy to administer and reliably scored (Valentini et al., 2015). Ideally, tests should also be sensitive at both ends of the distribution to be able to adequately discriminate levels of skillfulness in the motor domain (Sigmundsson et al., 2016) and able to assess the developmental process by measuring motor competence in different age groups with the use of the same test items in cross-sectional populations.

A growing number of studies have indicated that there are distinctions between performance on various measurements of motor competence, and that they should not be used interchangeably (Logan et al., 2014; Valentini et al., 2015; Rudd et al., 2016; Ré et al., 2018; Bardid et al., 2019). Logan et al. (2014) compared the TGMD-2 and MABC-2 in children 5 and 7 years old and found only low to moderate correlations between subscales and total performance on each assessment (r range between 0.27 and 0.52). Although most of the correlations were significant, the shared variance was low (*r*^2^ between 0.07 and 0.27) indicating weak practical significance. In addition, in this sample, the mean performance on the TGMD-2 was significantly lower (17th percentile) than the MABC-2 (42nd percentile), supporting the argument that different aspects of MC are measured by the two test batteries.

Similarly, Valentini et al. (2015), comparing children 4–10 years old on the TGMD-2 and MABC, found a low correlation between total performance on the two tests (*r* = 0.23) and higher levels of performance on the MABC compared to the TGMD-2 across all ages. Ré et al. (2018) compared the TGMD-2 and the Körperkoordinationstest für Kinder (KTK) in children between 5 and 10 years old and found only low-to-moderate correlations (r range between 0.34 and 0.52) between tests across age, suggesting that the two tests are measuring distinct aspects of MC. This finding was supported by Rudd et al. (2015) who showed, using confirmatory factor analysis, that the TGMD-2 and KTK are separate constructs in a hierarchical holistic model of MC. Ré et al. (2018) also showed that the TGMD-2 and KTK identified 39.4 and 18.4% of participants, respectively, with very low motor competence (percentile ≥ 5), adding to previous research that indicate a low agreement on ability to identify motor problems between different assessment tools (Crawford et al., 2001; Van Waevelde et al., 2007; Slater et al., 2010).

MC tests are made up of a test battery of motor tasks (items). The tasks in the battery are chosen to reflect skills within the framework of the test creator. Typically, there is one item (motor task) for each skill to be tested. The low pair-wise correlations of test batteries indicate that performance on one task is not related to performance on another task. Thus, low pair-wise correlations would provide evidence consistent with the specificity of abilities hypothesis (Henry, 1961a). In this long-standing view, skill acquisition is specific to the tasks in the context that they are learned (Magill and Anderson, 2014). That is, consistent with the specificity principle, performance in a task is skill specific. The principle of specificity of learning is supported by neuroscience (e.g., Edelman, 1992), rehabilitation (e.g., Kleim and Jones, 2008), and in cognitive tasks (e.g., Sigmundsson et al., 2013) and is also a key concept in constraint-based coaching (e.g., Renshaw and Holder, 2010). Overall, the concept of specificity suggest that learning is comparatively independent and specific. That is, learning a skill is associated with the strengthening of the neural network associated with that skill and increasing the likelihood of its execution in the future (Edelman, 1987, 1992). However, to date, relatively little research has examined the specificity principle in young children.

The present study had two aims. First, to investigate the association between a children specific MC test battery [Movement Assessment Battery for Children (MABC)] and a test battery which can be used across the lifespan (TMC) at both the overall test total level and individual test item level in a sample of primary school aged children (*X* = 7.9 years) to examine the specificity of motor abilities principle. Secondly, to explore relation of the MABC and TMC competence test structures. The MABC is a well-known test battery to examine children's motor competence; it is a validated, norm-referenced, and product-oriented assessment that quantitatively evaluates motor competence in children (Henderson and Sugden, 1992; Henderson et al., 2007). The TMC, on the other hand, is a relatively new assessment tool that examines two gross- and two fine-motor skills quantitatively with interval scale measures. Overall, comparison of assessment outcomes will provide extended knowledge to the research community as it relates to how different MC assessment capture different aspects of MC in human movement (Logan et al., 2017).

Based on previous findings with younger subjects (Haga et al., 2008) we expected low to moderate correlations between test-items within and between the two test batteries. A multivariate PCA was used to examine the multiple item relational structure of the two motor test batteries.

## Methods

### Participants

A total of 80 8-year-old children participated in the study (38 girls and 42 boys), the total population of a local selected school. The sample included children from a wide range of socio-economic backgrounds and reflected the population of children attending schools in this area. The mean chronological age was 7.9 years (SD 0.55).

### General Procedures

Full ethical review and approval was not required for this study in accordance with the national and institutional guidelines, however, the study was carried out in accordance with the recommendations of Norwegian Centre for Research Data and the Declaration of Helsinki. Written informed consent was obtained from the parents of all participants prior to the study commencement. Identification numbers were used to maintain data confidentiality.

The administration and scoring were carried out according to the instructions given in the two test manuals. Assessments of children were conducted in a quiet and appropriate room at the schools during normal school hours. Testing was conducted by instructors trained in the test protocol. All children were tested individually in a 1:1 setting, and the researcher explained and demonstrated each test. Verbal encouragement and support were provided throughout the testing procedure. The whole testing procedure lasted for about 25 min.

### Motor Assessments

The MABC was chosen as the standard test for comparison as it is frequently used in Europe and easy to administer (Henderson and Sugden, 1992). Additionally, the MABC provides objective, quantitative data on motor competence. The overall motor functioning of an individual is given through this broad test of tasks representative to those found in daily life, including fine and gross motor items. The TMC was designed to give an objective quantification of motor performance across the life span (Sigmundsson et al., 2016). However, the TMC results have not yet been compared with another test for motor competence in this age group only for adolescents (Gísladóttir et al., 2019).

### The Movement Assessment Battery for Children

MABC first edition was designed to identify children with motor co-ordination problems from ages 4 to 12 years. The test was developed by Henderson and Sugden (1992) and is an extended version of Test of Motor Impairment. It is a formal, standardized test and provides both a quantitative and a qualitative evaluation of the child's motor competence in daily life across a wide range of motor skills. Norms are provided for children aged 4–12 years. On the basis of these norms, it is possible to establish whether a child has normal motor performance (compared with 85% of children of the same age), borderline performance (85–95%) or, belongs to the 5% with a deviant performance (95–100%). In the age group 4–5 years, an MABC score of 10.5 would place the child at the 15th centile and a score >17.0 at the 5th centile.

MABC consists of three subtests, with a total of eight items, the content of which differs depending on the age range for which the test is used. In this study the items that were constructed for children aged 7–8 years were used. The subtests and items are: (i) manual dexterity, with the items placing pegs (measured: in seconds); Threading Lace (measured: in seconds) and Flower Trail (measured: number of errors); (ii) ball skills, with the items One-hand bounce and catch (measured: number of correctly executed catch out of 10 attempts) and Throwing bean Bag into Box (measured: number of successful throws out of 10 attempts); and (iii) balance, with the items Stork Balance (measured. number of seconds up to 20); Jumping in Squares (measured: number of correct and consecutive jumps (maximum of 5) and Heel—to-toe Walking (measured: number of correct consecutive steps the child takes; up to 15). On each item a score between 0 and 5 can be given, a higher score indicating worse performance. Item scores are summed to obtain scores on subtests. Scores on manual dexterity and balance range from 0 to 15, while scores on ball skills range from 0 to 10. Summation over the subtests results in a total score, which ranges from 0 to 40.

The MABC has a minimum test-retest reliability at any age of 0.75 and an interrater reliability of 0.70 (Henderson and Sugden, 1992; Tan et al., 2001). The MABC has been validated against other measures of motor performance, and low levels of agreement are reported between the MABC and BOTMP (Bruininks, 1978) in the identification of children with motor difficulties (<80%), as MABC identified more children with motor difficulties compared to the latter (Crawford et al., 2001; Slater et al., 2010). A comparison of convergent validity between the MABC and the Peabody Developmental Motor Scales (PDMS-2) showed a correlation of 0.76 between the total score of the two tests, but a low agreement between the ability to identify motor impairment (PDMS-2 was less sensitive than MABC to mild motor impairment) (Van Waevelde et al., 2007). Although the test has not yet been specifically standardized in Scandinavia, studies report that the norms provided in the MABC manual are valid for Scandinavian children (Sigmundsson and Rostoft, 2003). MABC has the same eight items as the MABC-2 for the 7–8 year old group.

### Test of Motor Competence

The TMC battery, consists of four different tests. Two fine motor tasks based on manual dexterity and two gross motor tasks based on dynamic balance. In all tasks, the performance measure is time to completion in seconds. The participants were given a practice run on all tasks. To quantify aspects of fine motor performance, the brick handling tasks: Placing Bricks (PB) and Building Bricks (BB) were conducted.

#### Placing Bricks

Eighteen square-shaped Duplo™ bricks are to be placed on a Duplo™ board (which has room for 3 × 6 bricks) as fast as possible. The participant is seated at a table and is given a practice run before the actual testing. The bricks were positioned in horizontal rows of three on the side of the active hand and the board was held firmly with the other hand. Both hands are tested.

#### Building Bricks

Twelve square-shaped Duplo™ bricks are used to build a “tower” as fast as possible. The participant holds one brick in one hand, and one brick in the other. At a signal, the participant assembled the bricks together one after one until all 12 have been put together to form a tower. Neither of the arms is allowed to rest on the table. The bricks should be held in the air all the time. The tasks were conducted with participants sitting comfortably at a table, and time was stopped when the participants released contact with the last brick. Brick handling has been used extensively in previous test batteries for motor performance (Yoon et al., 2006).

Gross motor tasks. Two test items were used to quantify aspects of gross motor performance: Heel to Toe Walking and Walking/Running in Slopes.

#### Heel to Toe Walking

This task is adapted from the tandem walking test (Rooks et al., 1997; Rinne et al., 2001) and is considered to be a measure of dynamic balance capabilities. Participants are required to walk along a straight line (4.5 m long) marked on the floor as fast as they can place their heel against the toes of the foot in each step.

#### Walking/Running in Slopes (W/R)

This task was an adaptation of the figure of eight test (Johansson and Jarnlo, 1991). The participant stands at the starting point and at a signal, the participant walks/runs as fast as possible in a figure of eight around two marked lines (1 m in width). Line 1 is 1 m from the starting point and Line 2 is 5.5 m from the starting point. If the participant starts to go on the right side of the Line 1, the subject will go to the left side of Line 2, turn around, and go back on the right side of Line 2 and left side Line 1, and over the starting point. The time is stopped when the participant arrives the starting point. Participants freely choose which direction they walk/run. The participants were wearing suitable shoes.

### Data Analysis

Task scores for TMC were transformed into standardized scores (z-scores) for the whole sample. A total test score of motor competence was calculated for each individual by taking the sum of the z-scores for the four tasks. Associations between tasks and test batteries total score were assessed by Pearson product moment correlation coefficients. All statistics were conducted with PASW statistics 19.0 (IBM, New York, US) with *p* < 0.05 as a statistical significance criterion. We also carried out a PCA analysis (with screeplot test) to determine the number of components to be extracted with an eigenvalue criterion larger than 1, and to analyze the relation between the collective items of the MABC and TMC.

## Results

The mean and standard deviation (SD) for the items of TMC and MABC are provided in Table 1.

**Table 1:** The mean and standard deviation (SD) for measures of tasks in TMC and MABC.

		**Mean**	**SD**	**Range**
TMC	Placing bricks	32.75	5.81	24.50–58.19
	Building bricks	18.48	3.46	12.22–29.25
	Heel-to-toe-walking	21.45	8.10	11.03–56.61
	Walking/running in slopes	6.83	1.06	5.19–12.26
Movement ABC	Placing pegs	4.19	1.04	0–5
	Threading lace	0.39	0.96	0–4
	Flower trail	0.90	0.13	0–5
	One hand bounce and catch	0.95	1.2	0–5
	Throwing bean bag into box	1.18	1.43	0–5
	Stork balance	0.58	1.12	0–5
	Jumping in squares	0.15	0.53	0–2
	Heal-to-toe walking	0.05	0.35	0–3

**Figure 1:**
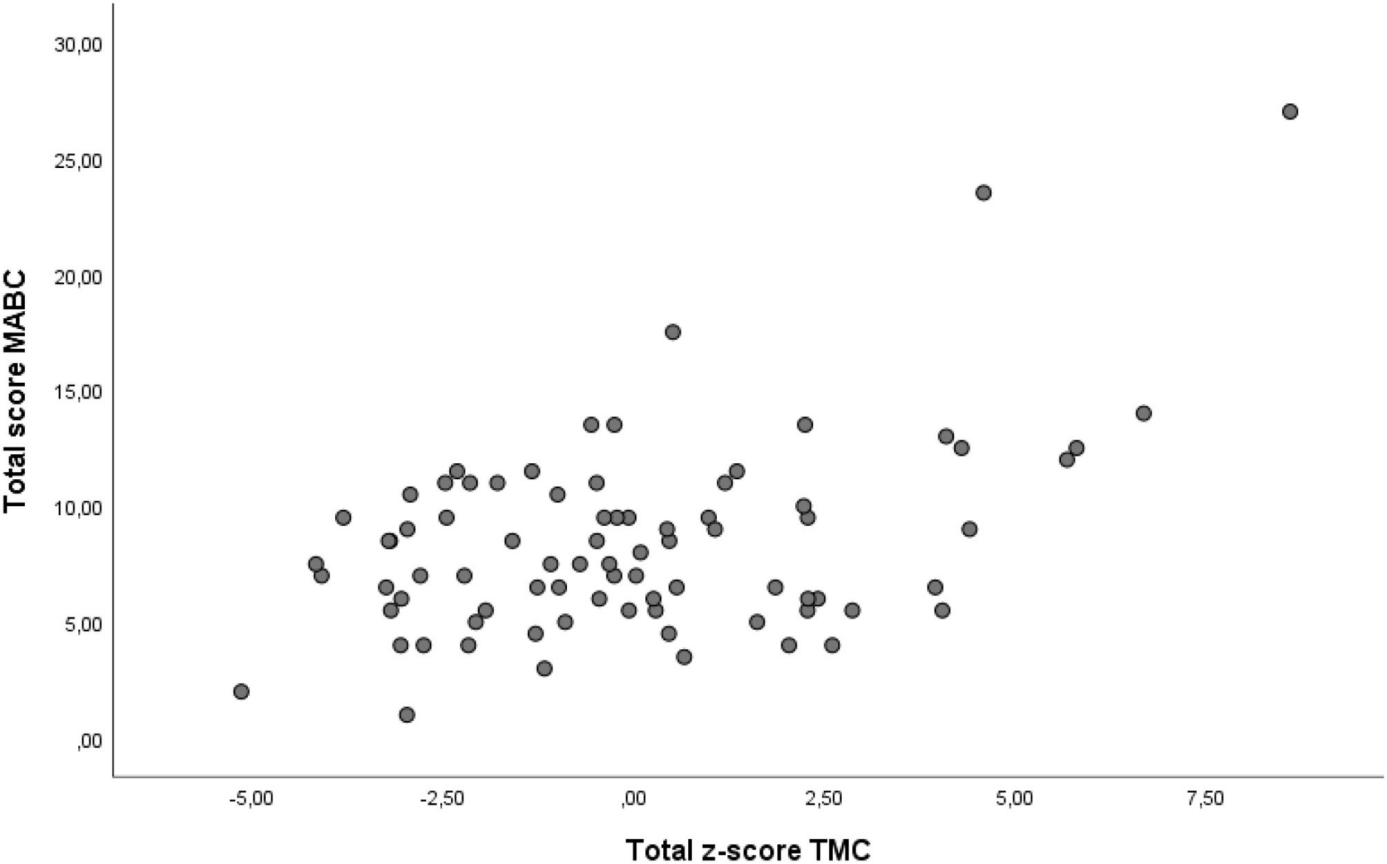
Scatterplot of the relationship across individual children between total score MABC and total z-score TMC.

Table 2 shows the intercorrelation between total score MABC and the four tasks and total z-score for TMC whereas Table 3 provides the intercorrelation between total z-score TMC and the eight tasks for MABC. Pearson product moment correlations between the eight subtasks of the MABC and the four subtasks of TMC are presented in Table 4. Correlation analysis between total score MABC and total z-score TMC was *r* = 0.46. Figure 1 shows the scatterplot of the relationship between total score MABC and total z-score TMC. Both MABC and TMC had Cronbach‘s Alpha value of 0.54.

**Table 2:** The inter-correlation between total score MABC and the four tasks from TMC (^*^P < 0.05; ^**^P < 0.01).

		**MABC total score**
TMC	Placing bricks	0.29^**^
	Building bricks	0.24^*^
	Heel-to-toe-walking	0.27^*^
	Walking/running in slopes	0.45^*^

**Table 3:** The intercorrelation between total score TMC and the eight tasks from MABC.

		**TMC total z score**
Movement ABC	Placing pegs	0.19
	Threading lace	0.11
	Flower trail	0.21
	One hand bounce and catch	0.33
	Throwing bean bag into box	0.30
	Stork balance	0.21
	Jumping in squares	0.28
	Heal-to-toe walking	0.24

**Table 4:** The intercorrelations (Pearsons) between performances in 12 motor tasks (*N* = 80).

		**2**	**3**	**4**	**5**	**6**	**7**	**8**	**9**	**10**	**11**	**12**
TMD	1. Placing bricks	0.44^**^	0.30^**^	0.02	0.06	0.18	0.15	0.17	0.19	0.07	0.19	0.24^*^
	2. Building bricks		0.39^**^	0.33^**^	0.07	−0.06	0.05	0.31^**^	0.20	0.06	0.15	0.19
	3. Heel-to-toe-walking			0.24^*^	0.11	0.21	0.03	0.14	0.18	0.11	0.18	0.23^*^
	4. Walkin/Running in Slopes				0.27^*^	−0.02	0.33^**^	0.28^*^	0.24^*^	0.30^**^	0.21	−0.020
Movement ABC	5. Placing pegs					0.06	0.10	−0.09	0.05	0.14	0.18	0.11
	6. Threading Lace						0.10	0.05	−0.03	0.14	0.18	0.20
	7. Flower Trail							0.24^*^	0.02	0.35^**^	0.20	0.12
	8. One hand bounce and catch								0.21	0.24^*^	0.25^*^	0.29^**^
	9. Throwing bean bag into box									0.20	0.09	−0.02
	10. Stork Balance										0.44^**^	0.37^**^
	11. Jumping in Squares											0.36^**^
	12. Heal-to-toe Walking											

** *Correlation is significant at the 0.01 level (2-tailed)*.

* *Correlation is significant at the 0.05 level (2-tailed)*.

In general, small to moderate correlations were found between the different tasks for both within and between test batteries. The highest moderate correlation was found between the TMC placing bricks and building bricks (*r* = 0.45) and the MABC the stork balance and jumping in squares (*r* = 0.45). The smallest correlation was found between MABC flower trail and throwing bean bag into box (*r* = 0.02) and between TMC placing bricks and walking/running in slopes (*r* = 0.02). The correlation between the similar task, but which applies a different approach for scoring, Heel-to-toe walking (TMC) (measured in sec used to cover a defined distance), and Heel-to toe walking (MABC) (measured in number of correct consecutive steps) was (*r* = 0.23). The same applies for similar tasks, measuring manual dexterity, Placing bricks and Placing pegs where the correlation was low (*r* = 0.06).

Principal components analysis (PCA) of the total 12 items indicated that a two-component solution based on eigenvalue criterion and screeplot test provided the best solution. Table 5 shows the variable weightings for the first two components. The first component (25%) was mostly comprised of high weighted items from MABC and the second component (12%) mainly comprised of TMC items.

**Table 5:** Variable weightings for the first 2 principal components.

**Motor test item**	**Component 1**	**Component 2**
MABC Stork balance	0.78	−0.18
MABC Jumping in squares	0.70	−0.27
MABC Heel–to-toe walking	0.59	−0.27
MABC Flower trail	0.59	−0.13
MABC Threading lace	0.38	−0.04
MABC Placing pegs	0.34	−0.11
TMC Building bricks	0.11	−0.82
TMC Heel–to–toe walking	0.22	−0.65
TMC Placing bricks	0.19	−0.64
MABC Throwing bean bag into box	0.10	−0.51
MABC One hand bounce and catch	0.42	−0.49
TMC Walking/running in slopes	0.43	−0.46

## Discussion

This study examined the specificity of motor abilities hypothesis (Henry, 1961a) in children aged 7–8 years old by examining the association between the MABC and TMC at the overall and item level. Secondly, the MABC and TMC factorial structure was explored. Findings supported the specificity hypothesis for our population suggesting that the two MC test batteries measured different aspect of MC.

The findings indicated moderate correlations between total score MABC and total z-score TMC sharing 21% of the variance. This is in line with the results from Gísladóttir et al. (2019) with a sample of adolescents in which the shared variance (18%) between the MABC-2 and TMC was of a similar magnitude as in the present study. The notion that there was only 21% shared variance between the MABC and TMC indicated that there was only moderate construct validity (Cronbach and Meehl, 1955). The finding of a modest total test correlations between the two tests was consistent with the PCA finding of low over all variance accounted for in the significant components and an orthogonal relation of the two tests in the first two components. Thus, the MABC and TMC appear to be tapping into different aspects of MC and appear largely independent providing support for the specificity hypothesis.

These findings are supported by other studies which have shown small to moderate associations between different MC test batteries for varied populations (Ré et al., 2018). The PCA supported this notion in finding a 2-component solution with the items from the MABC and TMC loading on a different specific component. A potential explanation for this is that the TMC is a criterion-referenced test which incorporates a continuum of skill while MABC is a norm-referenced test designated to identify children who are below a specific cutoff point. This is also supported by the correlation between heel to toe walking. Although both the MABC and TMC incorporates this skill it is scored differently and only showed a small correlation (*r* = 0.23). Although, a potential reason for this could be the notion that the MABC has a very narrow range of measurement (0–5) with a skewed distribution compared to TMC.

When looking at the relationship between the 12 different items from the two test batteries it can be seen that intercorrelations are low to moderate (r between 0.02 and 0.45) explaining only up to 20% of the shared variance. These findings are supported by previous research examining associations between different measures of motor skills in children. For example, Haga et al. (2008) found small correlations between different items within the MABC test battery in 4-year-old children. Drowatzky and Zuccato (1967) reported similar results when testing children aged 11–13 years on six different balance tasks with correlations ranging from 0.03 to 0.31.

The notion that small to moderate correlation exist between different items of a test battery is not surprising. Most motor test-batteries are designed to measure different aspects of motor behavior. High correlations in this instance would indicate that items might measure the same underlying motor construct indicating redundancy in the test-battery. However, the notion that items of different test batteries which pertain to measure similar aspects of motor behavior is problematic. For example, the pattern of correlations showed that both MABC and TMC measure heal-to-toe walking. However, the correlation between these two items was low (*r* = 0.23). This was due to the instructions provided to the participants to execute this task which in turn was related to the scoring of the task (time taking vs. number of steps with a maximum of 15). Future research should examine optimal ways of scoring items, be it based on quantitative aspects like time to completion, number of steps or the form of the movement and how this might be influenced by the instructions provided to execute the task.

The finding that highly similar motor tasks are low to moderately correlated does support the notion of specificity (Henry, 1961a; Magill and Anderson, 2014). In addition, test batteries that pertain to measure aspects of children's motor behavior appear to measure different aspects of motor competence. This is partially due to the way the behavior is measured (e.g., quantitative vs. qualitative) as well as the test used. For example, the MABC has a much smaller range of scoring which might result in ceiling effects reducing its discriminatory power. Similar findings of the significance of the method of measurement have been reported in the cognitive domain (Stöckel and Hughes, 2015; Sigmundsson et al., 2017). From a practical perspective this means that children should practice motor skills in a way that is representative to the domain they will execute the motor behavior. Such an approach is also in line with more contemporary approaches to sport coaching. For example, the constraint-based approach proposes that learning a motor skill is becoming adapted or attuned to the environment the skill is to be performed and not the development of an internal representation (e.g., Araujo and Davids, 2011).

Overall, the findings of the current study provide further support for the idea of specificity in individual's motor behavior (Henry, 1961b; Larkin and Parker, 2002). These findings on test item relations show that an individual can perform differently on different motor tasks (i.e., high and low respectively) even if the tasks are within the same category although scored differently as for example balance. Thus, motor competence consists of number of different skills with performance varying from one motor task to the next. Specificity has been observed in both the cognitive and motor domains, supporting the idea that learning is relatively task independent and specific and provide support a constraint-based approach to skill learning. However, future research should consider the scoring developed for different test batteries and explore their discriminatory power.

## Data Availability Statement

The raw data supporting the conclusions of this article will be made available by the authors, without undue reservation.

## Ethics Statement

Ethical review and approval was not required for the study on human participants in accordance with the local legislation and institutional requirements. Written informed consent to participate in this study was provided by the participants' legal guardian/next of kin.

## Author Contributions

All authors listed have made a substantial, direct and intellectual contribution to the work, and approved it for publication.

## Conflict of Interest

The authors declare that the research was conducted in the absence of any commercial or financial relationships that could be construed as a potential conflict of interest.
